# Effect of Host, Environment and Fungal Growth on Fungal Leaf Endophyte Communities in Taiwan

**DOI:** 10.3390/jof6040244

**Published:** 2020-10-23

**Authors:** Yu-Ling Huang

**Affiliations:** Department of Biology, National Museum of Natural Science, Taichung 40453, Taiwan; ylhuang@nmns.edu.tw

**Keywords:** community structure, elevation, gymnosperm, *Rhododendron*, vegetation

## Abstract

Fungal endophytes inhabit plant tissues without causing disease symptoms. They are highly diverse and distributed globally in all plants that have been investigated. Host, geographic, and environmental effects on endophyte communities have been reported in several studies, but the direct effect of fungal growth rate on endophyte composition has not been tested. To understand the relationship between foliar endophyte composition and fungal growth and to examine the effect of host, elevation, and climatic factors on the foliar endophyte communities, this study examined the foliar endophyte communities of representative gymnosperms and *Rhododendron* spp. across different elevations of Hehuanshan and Taipingshan forests in Taiwan. The isolation frequency and diversity of foliar endophytes were higher at low elevations than at high elevations. The foliar endophyte community structure differed as a function of host family and forest vegetation type. Elevation, mean annual temperature, and precipitation were significantly correlated with the community structure. Fungal growth rate was correlated with the endophyte abundance, which indicates that fast-growing fungi might have a competitive advantage when coexisting with other fungi in a plant host.

## 1. Introduction

Fungal endophytes are fungi that inhabit plants without causing disease symptoms. Several studies have reported the beneficial roles of symbiotic endophytes. For instance, endophytes can improve plant resistance to pathogens, herbivores, heat, and drought [1,2,3,4]. Additionally, some endophytes produce secondary metabolites that have potential for anti-cancer or biomedical applications [5]. Rodriguez et al. [6] defined four classes of endophytes based on their taxonomic position, transmission method, distribution, and diversity in plants. Class 3 endophytes are horizontally transmitted fungi that mostly inhabit the above-ground parts of woody plants. Compared to other classes of endophytes, class 3 endophytes are globally distributed and highly diverse in regions from low to high latitudes [7,8,9]. Multiple species of endophytes that coexist in a single plant form a unique community. Different plant hosts from different locations hold distinct endophyte communities. However, the factors and mechanisms that determine the assembly of endophyte communities are not fully understood. This study focused on class 3 endophytes (termed endophytes below) to investigate how host, environment, and fungal growth ability shape endophyte communities.

Diversification, dispersal, selection, and drift are the major processes of microbial community assembly, and each of them could affect the spatial or temporal patterns of a community [10]. Spore dispersal, host defense, and fungal proliferation are critical steps in the process of establishing foliar endophyte communities in tropical trees [11]. The dispersal ability of fungi is affected by the environment such as wind and precipitation [12], while host defense compounds alter the endophyte community assembly [13], and fungal proliferation is an outcome of niche competitions after colonization. Previous studies provide evidence that geographic locality and host identity are important factors in shaping foliar endophyte communities in gymnosperms at distances over thousand kilometers [14], and endophyte communities differ at a continental scale in diverse host plants and biogeographic areas [15]. Environmental gradients including temperature and rainfall are also determining factors in shaping foliar endophyte communities at a local scale [16]. In addition, the interaction types of the symbionts could affect the fungal communities more than abiotic factors [17]. All of these suggest that endophyte communities could be influenced by host, environment, and geographic factors, and the significance of which might vary depending on the scale or locality of studies. 

A recent study showed that the early colonization of endophytes in plants is an opportunistic process [18] though fungal proliferation after entering plant tissues has not been well studied. Intuitively, fast-growing microbes should be more abundant than slow-growing microbes in a community. The correlation between growth rate and abundance has been studied in bacteria. Krum et al. [19] showed that there was no significant relationship between the growth rate and abundance of bacteria in soil. However, fungi and bacteria have different proliferation strategies. The relationship between in vitro fungal growth rate and abundance in leaves has not been examined in fungal endophytes. Fast-growing endophytes might occupy more space in plants than slow-growing species, resulting in a high isolation frequency of fast-growing endophytes in the community.

Taiwan is a biodiversity hot spot located at the boundary of subtropical and tropical areas of the western Pacific coast. The unique geographic locality, climatic condition, and steep elevation gradients result in diverse vegetation types: from tropical forests at low elevation to subalpine zones at mountain tops above 3600 m [20]. The vertical distribution of vegetation in Taiwan is well classified [21] and a similar vertical pattern for associated endophyte communities in leaves could be predicted. This study hypothesized that (1) foliar endophyte communities change with the vegetation types, which is closely related to the elevation, and (2) fungal growth rate is related to the endophyte community composition. Specifically, endophyte diversity and abundance should decrease as the elevation increases, and phylogenetic composition and community structure should significantly differ between vegetation types. Additionally, fungal growth rates should be correlated with fungal abundance in a community.

## 2. Materials and Methods 

### 2.1. Sampling

**Figure 1:**
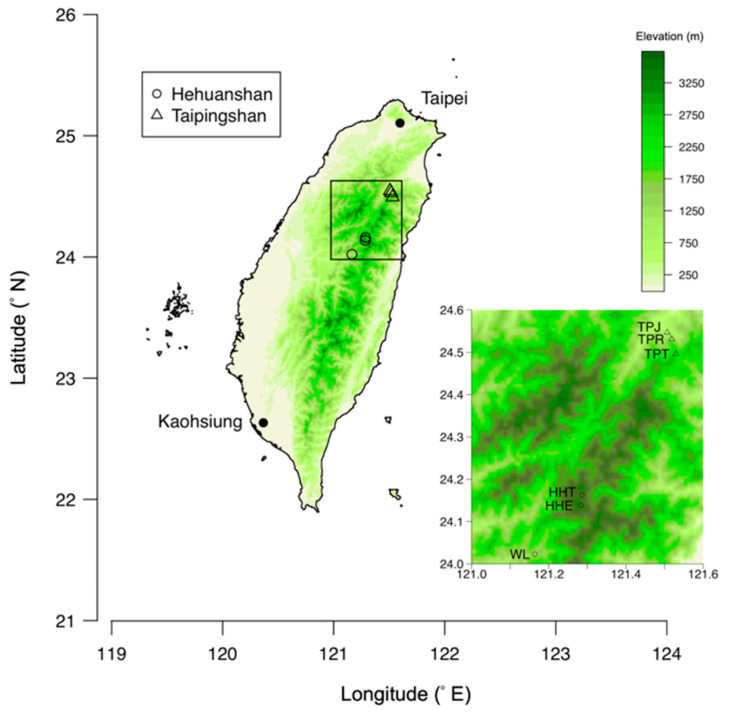
Map of sampling sites. There were six sampling sites at the Taipingshan and Hehuanshan areas in northeastern and central Taiwan. The major cities in Taiwan are indicated by black dots.

Healthy leaves were collected from two montane areas in central and northeast Taiwan, Hehuanshan and Taipingshan, respectively (Figure 1). For each montane area, two sample sites of the same vegetation at different elevations and one sample site of another vegetation at lower elevation were selected. The six sample sites were selected from altitudes 1100–3300 m at Hehuanshan and 450–2200 m at Taipingshan, respectively. The pure forests at the high elevation are composed of gymnosperm trees and often with *Rhododendron* species around. Since no single plant species can grow across ca. 2500 m elevational differences, at least one dominant gymnosperm species was paired with a nearby *Rhododendron* species at each site. Three trees of each species were sampled as replicates. The middle elevation area of Hehuanshan was not sampled due to the severe human destruction in the area. Occasionally, more than one gymnosperm species, or less than three trees of one species, were collected from a site according to changes in the forest vegetation along the trails and their availability. In total, thirty-five trees were sampled in March and April of 2018. Since the sampling processing after sampling was time-consuming, the sampling could not be done on the same day. However, all trees were sampled in spring to reduce the variations caused by sampling times. Three, ca. 30 cm long branches with leaves were collected from each tree, put into a Ziploc bag and transferred to the lab for processing within 72 h. Leaf branches of *Rhododendron* spp. were collected near the top of the shrubs; leaf branches of gymnosperms were cut at ca. 4 to 5 m above ground. The detailed information of sampling sites and trees is listed in Table 1 and Table S1.

### 2.2. Endophyte Isolation

Endophytes were isolated from surface-sterilized leaves. For *Rhododendron* spp., three leaves were selected from each of the three branches/tree, rinsed with tap water, and cut into 2 mm^2^ segments. For gymnosperms, leaves are not countable because of the needle or scale-like structure. Instead, three short branches about 10 cm long were selected from each of the three branches/tree and rinsed with tap water. All leaves from the 10 cm branches were cut into ca. 2 mm^2^ segments. The leaf segments from each tree were then pooled together and soaked in 95% ethanol for 10 s, 0.5% NaOCl for 2 min, and 70% ethanol for 2 min. After surface sterilization, fifty leaf segments from each tree were randomly selected and placed on a 2% malt extract agar (MEA) slant in individual 1.5 mL microtubes. The resultant 50 MEA slants with leaf segments from a single tree were then stored in a Ziploc bag and incubated at room temperature (between 22–30 °C) for 2 months. Each week, the slants were examined for fungal growth and, once observed, a small piece of mycelia was collected for DNA extraction (see below) and cultured onto two plates (6 cm in diameter) of 2% MEA. In total, 484 fungal endophytes were isolated from the 1750 leaf segments (50 leaf segments/tree × 35 trees). After a few months of growth, the cultured agar was cut into 8 mm cubes, stacked in a sterile 2 mL cryotube, and filled with double-autoclaved distilled water for preservation and further studies. The isolation frequency was calculated as the percent of isolates from each sample tree and was ln-transformed for normality.

### 2.3. DNA Extraction and Sequencing

Genomic DNA was extracted from fungal mycelia using the REDExtract-N-Amp^TM^ Plant Tissue PCR Kit (Sigma-Aldrich, MI, USA). Mycelia were first disrupted with Zirconium oxide beads (Next Advance Inc; NY, USA) in 100 µL of extraction buffer shaken at 30 times per second for 1 min using a TissueLyser II (QIAGEN Inc; MD, USA). After incubation at 95 °C for 10 min, 100 μL of dilution solution was added and gently mixed. The genomic DNA was stored at −20 °C until PCR amplification. ITS1F/LR3 primers (ITS1F: 5′-CTTGGTCATTTAGAGGAAGTAA-3′, LR3: 5′-GGTCCGTGTTTCAAGAC-3′) [22,23] were used to amplify the nuclear ribosomal DNA (nrDNA) fragment containing the partial small subunit (SSU), internal transcribed spacer 1 (ITS1), 5.8S, ITS2, and D1-D2 region of the large subunit (LSU). Each 20 μL PCR reaction mixture contained 0.8 μL of each primer 10 μM, 1 μL genomic DNA, 10 μL of REDExtract-N-Amp^TM^ PCR ReadyMix, and 7.4 μL of sterile distilled water. To confirm PCR success, PCR products were loaded in a 1% agarose gel in 1X TAE buffer with 5% HealthView nucleic acid stain (Genomics, Taipei, Taiwan) and run for 25 min at 100V. Gels were visualized using a G:Box Mini with GeneSys software (SYNGENE, Frederick, MD, USA). If the amplification with ITS1F/LR3 primers was unsuccessful, ITS1F/ITS4 primers (ITS4: 5′-TCCTCCGCTTATTGATATGC-3′) [24] were used instead. 

The PCR products were diluted with 5 μL of sterile distilled water and cleaned with 1 μL of ExoSAP-IT PCR Product Cleanup Reagent (Thermo Fisher Scientific Inc; Waltham, MA, USA) at 37 °C for 1 h followed by another incubation at 80 °C for 15 min. For each sample, 6 μL of cleaned PCR product and 1 μL of 10 μM primer were sent to the DNA Sequencing Core, College of Medicine, National Taiwan University for Sanger sequencing by ABI 3730 xl DNA Analyzer (Thermo Fisher Scientific Inc; Waltham, MA, USA) with BigDye Terminator v3 Cycle Sequencing Kits (Thermo Fisher Scientific Inc; Waltham, MA, USA). Each isolate was sequenced bidirectionally, and both sequences were assembled with Phred and Phrap [25,26] in Mesquite version 3.5 [27]. Assembled sequences were edited manually using Chromaseq Package version 1.31 [28]. Overall, 481 of the 484 fungal isolates were successfully sequenced. The DNA sequences generated and analyzed during the current study are available in the GenBank with accession numbers MT183700 to MT184172.

### 2.4. OTU Clustering

All sequences were clustered into operational taxonomic units (OTUs) using the “sanger_otu_cultering_workflow” on the Mobyle SNAP Workbench [29,30]. The workflow selected and trimmed sequences containing ITS1-5.8S-ITS2, removed the chimera sequences, and created a distance matrix for OTU clustering. OTU clustering was based on 95% sequence similarity using the furthest neighbor algorithm in mothur [31,32]. Overall, 473 sequences were used in OTU clustering, resulting in 94 OTUs (Table S2). This OTU matrix was used to calculate Fisher’s alpha, which represents the diversity of foliar endophytes in each tree.

### 2.5. Phylogenetic Placement

To determine the phylogenetic placement of each isolate at class level, all sequences were submitted to the Tree-Based Alignment Selector toolkit (T-BAS) [33,34] for automatic alignment and phylogenetic analysis against other Pezizomycotina in the database. The results were used to examine the overall phylogenetic composition at the class level.

### 2.6. Fungal Growth

Representative isolates of the 94 OTUs were individually cultured on a 6 cm plate of 2% MEA. Mycelia of most isolates covered the whole plate within 4 to 10 days. Slow-growing isolates were grown until the radius of colony reached 1 cm before transfer. A 4 mm agar disc containing mycelia cut from the edge of colony was placed on a 9 cm plate of 2% MEA. The colony radius was measured 7 days after transfer (Table S2). The radius of a single colony of each representative isolate was measured without replicates due to the limitations that each OTU had a single representative isolate, and subcultures of the isolates could not represent real biological replicates.

### 2.7. Data Analysis

Multiple regression models were built to analyze the effects of host family and elevation on isolation frequency and diversity. Effect of host was tested at family level because host specificity of endophytes is mostly confined to the family level, not genus or species levels [35]. Samples were split into low (<1500 m), middle (1500–2500 m), and high elevation (>2500 m) groups according to Chen [36], but the boundary between low and middle elevation was adjusted to 1200 m in Taipingshan because of the higher latitude. Analysis of variance (ANOVA) was used to test the effect of each factor in the multiple regression model. An OTU matrix was created based on the OTU clustering results, which used OTUs isolated from each sample tree as an individual community of foliar endophytes. The OTU matrix was then used for community structure analyses after removing singletons and outliers, which included 21 communities (trees). All communities from the sites higher than 3000 m were excluded due to low isolate counts. The community structure was visualized by non-metric multidimensional scaling (NMDS), and the associated statistical analysis was calculated by permutational multivariate analysis of variance (PERMANOVA) to test the effects of host family and vegetation types to the community structure. The Jaccard index and the Morisita–Horn index were used to calculate the dissimilarity of community structure. Climatic data used in the analyses were acquired from WorldClim Version 2 [37]. The classification of forest vegetation followed [21]. Elevation and climatic data were fit to the NMDS surface using generalized additive models. Fisher’s exact test was used to test whether the class-level compositions were the same among vegetation types. 

To test if the leaf endophyte composition of vegetation correlated with environmental factors, fungal class dissimilarity and environmental dissimilarity were calculated and their correlation tested with a Mantel test. The Morisita–Horn index was used to estimate the dissimilarity of class-level composition between vegetation types. Environmental dissimilarity was calculated as the Euclidean distance of factor scores from principal component analyses that included the annual mean temperature, mean diurnal temperature range, annual precipitation, precipitation seasonality, and elevation. In addition, multiple regression on distant matrices (MRM) was used to test the correlation between the community composition of foliar endophytes and climatic factors. 

To examine the correlation between fungal growth and the composition of leaf endophyte communities, a bootstrap Kolmogorov–Smirnov test (K-S test) was used to test the similarity between fungal growth distributions and OTU abundance in a sample tree. The fungal colony radius on day 7 represented the fungal growth of an OTU; the number of isolates of each OTU represented the OTU abundance. Sample trees with more than one OTU were selected for analysis. In each tree, singleton OTUs were excluded from the analyses.

All the statistical analyses and the climatic data acquisition were conducted in R version 3.5.2 using vegan, ecodist, Matching, and raster packages.

## 3. Results

This study isolated 484 endophytes from 1750 leaf segments of 35 host plants at 6 sample sites in Hehuanshan and Taipingshan. The overall isolation frequency was 27.7%, which was calculated as the percentage of the total number of isolates to the total number of leaf segments. OTU clustering based on 95% sequence similarity resulted in 94 OTUs (Table S2). Phylogenetic placement identified all isolates as Ascomycota. Sordariomycetes was the most dominant class, which included 83.4% of the sequenced isolates. Other identified classes were Dothideomycetes (10.6%), Eurotiomycetes (4.2%), Lecanoromycetes (1.7%), and Leotiomycetes (0.2%).

### 3.1. Isolation Frequency and Diversity Differed as a Function of Host Family and Elevation

**Figure 2:**
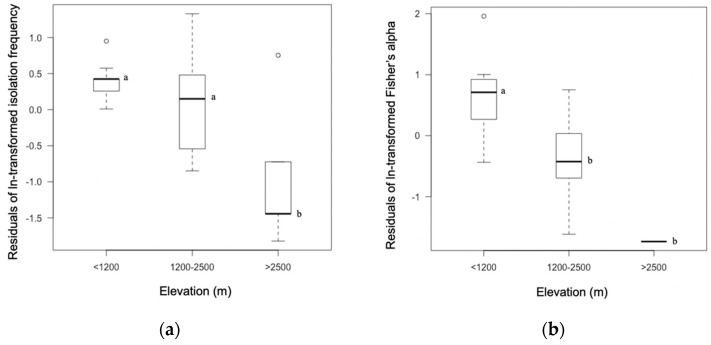
Isolation frequency and diversity differed as a function of elevation. (**a**) Isolation frequency. (**b**) Diversity. Residuals of ln-transformed isolation frequency or Fisher’s alpha were used to exclude the effect of host family. Tukey’s HSD test was used for the post hoc pairwise comparison. Lowercase letters in each graph indicate the significant differences tested by Tukey’s HSD test. Cycles are outliers.

Endophytes were isolated from 27 of the 35 sample trees. The isolation frequency of each tree represented the abundance of foliar endophytes in the community (Table S1) and was high at the elevation lower than 2500 m when the effect of host family was excluded (Figure 2). A multiple regression model built with the isolation frequency to test the effects of host family, elevation, and montane area showed these factors together explained 80.7% of variations in isolation frequency (R^2^ = 0.8073, F_(5, 21)_ = 17.6, *p* < 0.001). Individually, host family and elevation were significant factors, while montane area was not (Table 2).

Fisher’s alpha was used to calculate the leaf endophyte diversity of each host tree (Table S1), which was also high at lower elevations when the effect of host family was excluded (Figure 2). A multiple regression model indicated that host family, elevation, and area together explained 81.0% of the variation in endophyte diversity (R^2^ = 0.8098, F_(5,15)_ = 12.8, *p* < 0.001). The effects of host family, elevation, and area were tested by ANOVA, and the results showed that leaf endophyte diversity differed as functions of host family and elevation (Table 2).

### 3.2. Host Family, Forest Vegetation, and Climatic Factors Shape Endophyte Community Structures

**Figure 3:**
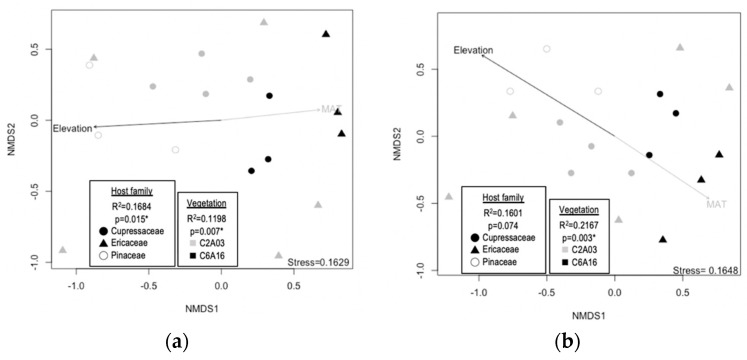
NMDS visualization of community structure using the Taipingshan data set. (**a**) Jaccard and (**b**) Morisita–Horn index of dissimilarity was calculated for the analyses, respectively. Each spot represents the endophyte community structure in a sample tree. The colors represent the vegetation types coded in Li et al. [21] (C2A03: *Chamaecyparis* montane mixed cloud forest; C6A16: *Zelkova–Quercus* rock-outcrop forest). Asterisks indicate the significance of PERMANOVA tests. Arrows indicate the significant relationships of elevation (Jaccard index: R^2^ = 0.5914, *p* = 0.004; Morisita–Horn index: R^2^ = 0.6384, *p* = 0.002), mean annual temperature (MAT; Jaccard index: R^2^ = 0.6255, *p* = 0.001; Morisita–Horn index: R^2^ = 0.6827, *p* = 0.001), and annual precipitation (Jaccard: R^2^ = 0.7038, *p* = 0.001; Morisita–Horn: R^2^ = 0.7625, *p* = 0.02) with the community structure. The annual precipitation vector is not shown due to overlap with elevation.

Unlike the isolation frequency and diversity analyses, the geographic area was a significant factor in shaping community structures. To exclude the effect of geographic variations while testing the effects of host family and vegetation, only data from Taipingshan were used for community structure analyses. Leaf endophyte community structures differed as a function of host family and vegetation type when using the Jaccard index but only differed as a function of vegetation type when using the Morisita–Horn index (Figure 3). Moreover, fitting the mean annual temperature, annual precipitation, and elevation to the NMDS surface indicated that community structures were significantly correlated with these climatic factors (Figure 3).

### 3.3. Phylogenetic Composition Differed among Vegetation Types and Was Correlated with Environmental Factors

**Figure 4:**
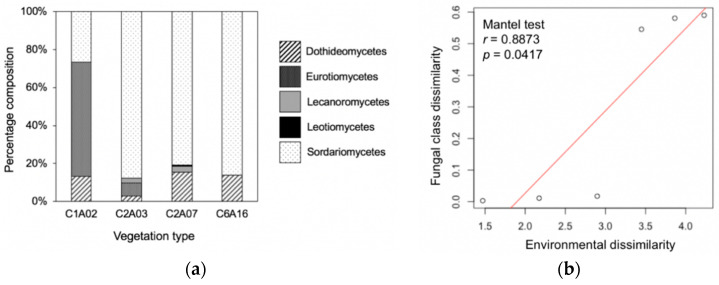
The class-level phylogenetic composition of endophytes differed among vegetation types. (**a**) Phylogenetic compositions of vegetation type. (**b**) The correlation of fungal class dissimilarity with environment. The fungal class dissimilarity (Morisita–Horn index) between vegetation types was significantly correlated with environmental dissimilarity.

The phylogenetic composition at the class level reflected the different compositions of leaf endophyte communities between forest vegetations (Figure 4). Fisher’s exact tests confirmed that phylogenetic composition was significantly associated with forest vegetation (*p* < 0.001). Additionally, the dissimilarity of class-level composition between vegetation types was significantly correlated with the environmental dissimilarity (Figure 4). MRM indicated that class-level dissimilarity was also significantly correlated with the annual temperature range (*p* = 0.014) and precipitation seasonality (*p* = 0.009). These results suggest that environmental factors could shape the endophyte community in different vegetation types.

### 3.4. Leaf Endophyte Composition Was Correlated with Fungal Growth

The distributions of fungal growth and OTU abundance in sample trees are shown in Figures S1 and S2. K-S test showed the distributions of fungal growth and OTU abundance were significantly correlated for 15 of the 17 trees (Table 3). These results suggest that the composition of leaf endophyte communities was approximately correlated with the rate of fungal growth.

## 4. Discussion

This study examined the leaf endophyte diversity of representative gymnosperms and *Rhododendron* spp. across different elevations in the Hehuanshan and Taipingshan forests of Taiwan. Both isolation frequency and diversity decreased as the elevation increased. Community structures differed as a function of host family and forest vegetation. The preliminary phylogenetic analysis indicated different phylogenetic compositions between forest vegetation types. Environmental factors were also correlated with community structure and composition. In addition, fungal growth was a critical factor for leaf endophyte community composition.

Communities changing with elevation has been reported in macroorganisms, while the diversity of some microorganisms has no clear association with the elevation gradient [38,39,40]. Studies including mycorrhiza and macrofungi report correlations between fungal diversity and elevation gradients [41,42,43]. However, the fungal assemblages in leaves and roots do not follow similar elevational diversity patterns [44]. A recent study of endophytes in *Pinus ponderosa* along an elevation gradient at the southwestern United States shows a trend for higher abundances of foliar endophytes at low elevations [45]; an illumina-sequencing analysis of endophytes also showed the tendency for diversity to decrease as elevation increases [46]. In the present study, the abundance and diversity of foliar endophytes were higher at low elevations and lower at high elevation, which is consistent with the hypothesis of this study. Although the differences detected in this study could be due to host effects, the multiple regression analyses and ANOVA supported that both host family and elevation explained the variations in the leaf endophyte abundance and diversity. 

Endophytes are horizontally transmitted between plants through spores dispersed in the air [11]. While endophytes residing in plant tissues might not be affected by the outer environment, the environment could affect spore dispersal patterns and the composition of host plant communities. The abundance of airborne fungal spores is reported to be associated with both temperature and relative humidity [47]. Additionally, interactions between endophytes and host plants could determine the number or species of endophytes that can colonize the plants. Such interaction includes that host plants produce defense compounds against fungi and thus alter the assembly of endophyte communities [13]. Both dispersal and the host species identity could limit endophyte colonization, thereby determining the assembly of endophyte communities [48]. In this study, the abundance, diversity, and structure of leaf endophyte communities differed as a function of host family, and the structure and composition of leaf endophyte communities were related to environmental factors. Together, these data support the idea that the environment and host are important factors in shaping leaf endophyte communities.

Vegetation integrates the effects of host and environment at a specific elevation. In general, temperature decreases and precipitation increases along with the elevation, though precipitation may differ by area. This study was unable to separate the effect of elevation from precipitation or temperature due to the collinearity between elevation and the climatic factors, but the results still indicated the integrated effects of environmental factors on leaf endophyte communities. In addition, the changes in the climatic factors alter the plant composition in the forest; thus, forest vegetation and elevation are highly correlated [21,49]. However, two sites found at the same elevation, but at different latitudes and geographic areas, could have very different ecosystems. In contrast, the vegetation reflects geographic and environmental factors as well as plant compositions. All these are critical factors in determining endophyte communities. The results of this study showed that the composition of leaf endophyte communities differed by vegetation types, and that the dissimilarity of leaf endophyte composition increased along with the dissimilarity of environment between vegetation types. Together, these data suggest a connection between vegetation types and endophyte communities in leaves. 

Recently, high-throughput sequencing has been widely used in microbial ecology to study the environmental samples. Johnston et al. [50] compared leaf fungal diversity in Nothofagaceae forests using culturing and high-throughput sequencing, and the results showed culture-based method might underestimate fungal diversity as compared to high-throughput sequencing method, while both methods could detect the effects of host and sites on community structures. The aims of this study were to examine the effects of host and environment on leaf endophyte communities and to study the relationships of intrinsic growth rate with endophyte communities. Although high-throughput sequencing could detect higher diversity, it could not preserve the individual fungal species for growing test. One advantage of culture-based method is to preserve the living fungal cultures which are valuable for further studies. Fungal endophytes produce secondary metabolites, which might be useful for biomedical application [51], and living cultures from ecological surveys are excellent sources for further studies. Moreover, the cryptic biodiversity of fungi has not been fully studied yet, and the preserved cultures from the community studies provide the chance for discovering new taxa. Using the 94 representative isolates to conduct BLAST queries found 3 OTUs did not match any species names in the GenBank, and the sequence identities of 23 OTUs with species named hits were lower than 95%. The species names of the top hits were used to search the fungal databases in Taiwan, which included the fungal herbarium of National Museum of Nature Science (TNM), the List of the Fungi in Taiwan, and the strain collection catalog of Bioresource Collection and Research Center (BCRC). Thirty-nine OTUs had no matched species in the three databases. Although the databases do not include all known fungal taxa in Taiwan, the preliminary searches provide the possibility of discovering new or new-recorded species in Taiwan. 

An endophyte community consists of multiple fungal species from a regional species pool, and the host and environment are external filters that affect endophyte community assembly [52]. After filtering by these external factors, fungi grow and compete with each other until a balanced community is reached. Therefore, the intrinsic fungal growth ability might determine the assembly of endophyte communities. The distribution of endophyte growth was approximately consistent with the OTU abundance of selected communities in this study. This suggests that frequently isolated species are fast-growing endophytes and the rare species are slow-growing endophytes. In bacterial communities, the growth rate in monoculture is correlated with the competitive ability, and simple competition rules can predict the assembly of bacteria [53]. To the best of my knowledge, this is the first study to test the relationship between fungal growth rate in culture and the OTU abundance in endophyte communities. The distributions of fungal growth and OTU abundance are not perfectly correlated because endophyte communities are not exclusively determined by the growth ability of fungi. Interspecific interaction with the host plants and competition with other fungi could determine the communities as well. Further studies are necessary to examine whether fast-growing endophytes have a better competitive ability than slow-growing endophytes and to discover the relationships between interspecific competitions and endophyte community assembly. 

## 5. Conclusions

This study examined the effects of host, elevation, and climatic factors on leaf endophyte communities. Vegetation integrated the effects of these factors and was related to endophyte compositions. The intrinsic fungal growth rate was correlated with OTU abundance in the communities, which indicated that fast-growing fungi might have a competitive advantage when coexisting with other fungi in a plant host. These findings add information to our understanding of endophyte communities in Taiwan and provide evidence for the importance of host, environment and fungal growth in endophyte community assembly.

## Figures and Tables

**Table 1:** Sampling site information and tree species collected at each site.

Site	Area	Coordinates (°)	Altitude (m)	Vegetation *	Species ^+^ Collected (the Number of Trees Sampled)
HHE	Hehuanshan	24.139, 121.283	3126–3297	C1A02	*Abies kawakami* (3) *Rhododendron pseudochrysanthum* (3)
HHT	Hehuanshan	24.162, 121.287	2998–3004	C1A02	*Tsuga chinensis* (3)
WL	Hehuanshan	24.023, 121.64	1086–1130	C2A07	*Cunninghamia lanceolate* (3)
TPT	Taipingshan	24.496, 121.530	1960–2183	C2A03	*Tsuga chinensis* (3) *Chamaecyparis formosensis* (1) *Chamaecyparis obtuse* var. *formosana* (2) *Rhododendron formosanum* (3)
TPR	Taipingshan	24.531, 121.518	1306–1471	C2A03	*Chamaecyparis formosensis* (1) *Rhododendron mucronatum* (3)
TPJ	Taipingshan	24.547, 121.507	463–563	C6A16	*Calocedrus formosana* (3) *Rhododendron mucronatum* (3)

* C1A02: *Abies–Tsuga* upper-montane coniferous forest; C2A03: *Chamaecyparis* montane mixed cloud forest; C2A07: *Phoebe–Machilus* sub-montane evergreen broad-leaved forest. C6A16: *Zelkova–Quercus* rock-outcrop forest [21]. ^+^ Trees collected in this study are evergreen trees; family of each species is listed in Table S1.

**Table 2:** ANOVA table of the multiple regression model for isolation frequency and diversity.

Factors	Df	Sum of Squares	Mean Square	F Value	*p* Value
(a) Isolation frequency					
Host family	2	20.9343	10.4671	31.093	<0.0001 *
Elevation	2	8.6670	4.3335	12.873	0.0002 *
Area	1	0.0246	0.0246	0.073	0.7897
Residuals	21	7.0695	0.3366		
(b) Diversity					
Host family	2	7.1871	3.5935	11.974	0.0008 *
Elevation	2	11.8660	5.9330	19.770	0.0001 *
Area	1	0.1129	0.1129	0.376	0.5489
Residuals	15	4.5016	0.3001		

Df: degree of freedom; * indicates the significance.

**Table 3:** Bootstrap K-S test compared the distributions of fungal growth and OTU abundance in the investigated sample trees.

Host Species	Tree Code	Number of OTUs	D	*p* Value ^+^
*Chamaecyparis obtuse var*. *formosana*	TP01CO	3	1.000	0.059
	TP02CO	2	0.500	0.889
*Chamaecyparis formosensis*	TP03CF	3	0.667	0.440
	TP07CF	2	1.000	0.201
*Calocedrus formosana*	TP14CaF	7	0.571	0.143
	TP15CaF	5	0.600	0.245
	TP16CaF	7	0.714	0.035 *
*Cunninghamia lanceolate*	WL01CL	5	0.600	0.265
	WL02CL	8	0.500	0.208
	WL03CL	8	0.625	0.049 *
*Rhododendron mucronatum*	TP07RM	2	1.000	0.217
	TP11RM	3	0.333	0.963
	TP12RM	4	0.250	0.964
*Rhododendron formosanum*	TP01RF	2	1.000	0.167
	TP04RF	2	0.500	0.893
*Tsuga chinensis*	TP01TC	3	0.667	0.408
	TP03TC	2	0.500	0.890

^+^ Bootstrap *p* value. * indicates the significant value (*p* < 0.05) to reject H_0_ (H_0_: two distributions are the same).

## Supplementary Materials

The following are available online at https://www.mdpi.com/2309-608X/6/4/244/s1. Table S1: List of host trees sampled in this study. Table S2: Isolate list and 7-day fungal growth of 94 OTUs.
