# Supplementary material for: Effect of Host, Environment and Fungal Growth on Fungal Leaf Endophyte Communities in Taiwan
Source: J Fungi (Basel). 2020 Oct 23;6(4):244. doi: 10.3390/jof6040244 (PMC7712724; doi:10.3390/jof6040244)
Supplement: Supplementary file 1 [file jof-06-00244-s001.zip › Supplementary Files/Figure S2.docx]

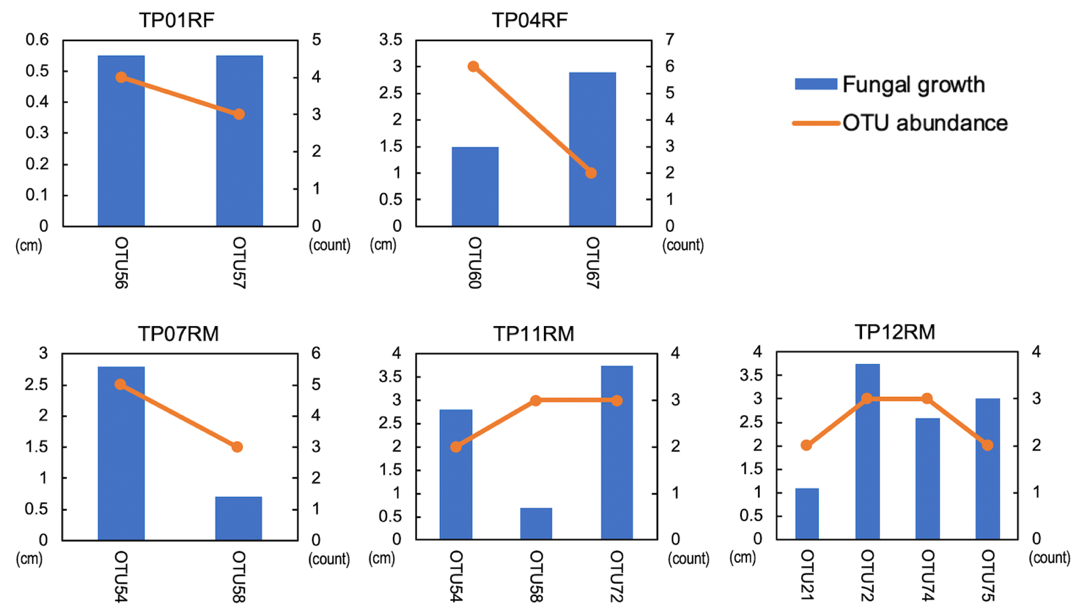


**Figure S2.** The distributions of fungal growth and OTU abundance in *Rhododendron* spp. Fungal growth is the 7-day colony radius of the representative isolate of the OTU. OTU abundance is the number of isolates of the OTU in the sample tree. Singleton OTUs in each tree was excluded from the analyses.
