# Supplementary material for: Effect of Host, Environment and Fungal Growth on Fungal Leaf Endophyte Communities in Taiwan
Source: J Fungi (Basel). 2020 Oct 23;6(4):244. doi: 10.3390/jof6040244 (PMC7712724; doi:10.3390/jof6040244)
Supplement: Supplementary file 1 [file jof-06-00244-s001.zip › Supplementary Files/Table S1.docx]

**Table S1.** List of host trees sampled in this study.

| Tree Code | Site | Family | Species | Isolation frequency ( %) | Fisher’s α |
| --- | --- | --- | --- | --- | --- |
| HH01RP | HHE | Ericaceae | *Rhododendron pseudochrysanthum* | 0 | NA |
| HH01JS | HHE | Cupressaceae | *Juniperus squamata* | 0 | NA |
| HH02RP | HHE | Ericaceae | *Rhododendron pseudochrysanthum* | 0 | NA |
| HH02JS | HHE | Cupressaceae | *Juniperus squamata* | 0 | NA |
| HH03RP | HHE | Ericaceae | *Rhododendron pseudochrysanthum* | 2 | >100 |
| HH03JS | HHE | Cupressaceae | *Juniperus squamata* | 0 | NA |
| HH04RP | HHE | Ericaceae | *Rhododendron pseudochrysanthum* | 6 | >100 |
| HH04AK | HHE | Pinaceae | *Abies kawakamii* | 0 | NA |
| HH05AK | HHE | Pinaceae | *Abies kawakamii* | 18 | 0.29 |
| HH06AK | HHE | Pinaceae | *Abies kawakamii* | 0 | NA |
| HH07TC | HHT | Pinaceae | *Tsuga chinensis* | 2 | >100 |
| HH08TC | HHT | Pinaceae | *Tsuga chinensis* | 0 | NA |
| HH09TC | HHT | Pinaceae | *Tsuga chinensis* | 2 | >100 |
| TP01TC | TPT | Pinaceae | *Tsuga chinensis* | 20 | 2.76 |
| TP01CO | TPT | Cupressaceae | *Chamaecyparis obtusa* var. *formosana* | 38 | 5.21 |
| TP01RF | TPT | Ericaceae | *Rhododendron formosanum* | 16 | 1.74 |
| TP02TC | TPT | Pinaceae | *Tsuga chinensis* | 8 | 2.62 |
| TP02CO | TPT | Cupressaceae | *Chamaecyparis obtusa* var. *formosana* | 38 | 4.00 |
| TP03TC | TPT | Pinaceae | *Tsuga chinensis* | 32 | 3.49 |
| TP03RF | TPT | Ericaceae | *Rhododendron formosanum* | 20 | 0.75 |
| TP03CF | TPT | Cupressaceae | *Chamaecyparis formosensis* | 76 | 3.73 |
| TP04RF | TPT | Ericaceae | *Rhododendron formosanum* | 20 | 2.47 |
| TP05RM | TPR | Ericaceae | *Rhododendron mucronatum* | 6 | >100 |
| TP06RM | TPR | Ericaceae | *Rhododendron mucronatum* | 10 | >100 |
| TP07RM | TPR | Ericaceae | *Rhododendron mucronatum* | 20 | 2.47 |
| TP07CF | TPR | Cupressaceae | *Chamaecyparis formosensis* | 28 | 3.98 |
| TPJ1RM | TPJ | Ericaceae | *Rhododendron mucronatum* | 22 | 5.4 |
| TPJ2RM | TPJ | Ericaceae | *Rhododendron mucronatum* | 32 | 9.5 |
| TPJ3RM | TPJ | Ericaceae | *Rhododendron mucronatum* | 16 | 26.78 |
| TPJ4CaF | TPJ | Cupressaceae | *Calocedrus formosana* | 100 | 15.72 |
| TPJ5CaF | TPJ | Cupressaceae | *Calocedrus formosana* | 66 | 4.99 |
| TPJ6CaF | TPJ | Cupressaceae | *Calocedrus formosana* | 98 | 8.27 |
| WL01CL | WL | Cupressaceae | *Cunninghamia lanceolata* | 72 | 17.77 |
| WL02CL | WL | Cupressaceae | *Cunninghamia lanceolata* | 100 | 10.09 |
| WL03CL | WL | Cupressaceae | *Cunninghamia lanceolata* | 100 | 21.04 |

Isolation frequency was calculated as the percentage of numbers of isolates. Fisher’s α larger than 100 were excluded for ecological analyses. NA: non-applicable due to zero isolate.
