# Supplementary material for: Effect of Host, Environment and Fungal Growth on Fungal Leaf Endophyte Communities in Taiwan
Source: J Fungi (Basel). 2020 Oct 23;6(4):244. doi: 10.3390/jof6040244 (PMC7712724; doi:10.3390/jof6040244)
Supplement: Supplementary file 1 [file jof-06-00244-s001.zip › Supplementary Files/Table S2-revision.docx]

**Table S2.** Isolate list and 7-day fungal growth of 94 OTUs.

| OTU | Representative Isolate (Genbank accession no.) | Colony radius (cm) | Isolates |
| --- | --- | --- | --- |
| OTU1 | 18WL001 (MT183700) | 3.8 | 18WL001 |
| OTU2 | 18WL026 (MT183723) | 4.05 | 18WL026, 18WL007, 18WL002 |
| OTU3 | 18WL070 (MT183765) | 1.85 | 18WL070, 18WL003 |
| OTU4 | 18WL053 (MT183748) | 3.3 | 18WL115, 18WL113, 18WL100, 18WL090, 18WL108, 18WL083, 18WL131, 18WL052, 18WL053, 18WL004 |
| OTU5 | 18WL114 (MT183808) | 3.6 | 18TP479, 18TP474, 18TP464, 18TP426, 18TP461, 18TP388, 18WL086, 18WL050, 18WL061, 18WL118, 18WL039, 18WL058, 18WL076, 18WL040, 18WL114, 18WL018, 18WL068, 18WL051, 18WL014, 18WL021, 18WL047, 18WL082, 18WL081, 18WL056, 18WL060, 18WL005 |
| OTU6 | 18WL119 (MT183813) | 0.8 | 18WL064, 18WL117, 18WL034, 18TP019, 18WL102, 18WL032, 18WL031, 18WL029, 18WL088, 18WL030, 18WL024, 18WL016, 18WL110, 18WL011, 18WL119, 18WL089, 18WL006 |
| OTU7 | 18WL008 (MT183707) | 2.85 | 18WL008 |
| OTU8 | 18TP481 (MT184145) | 1.2 | 18TP028, 18TP008, 18TP004, 18TP003, 18TP001, 18TP481, 18TP410, 18TP384, 18WL020, 18WL009 |
| OTU9 | 18WL010 (MT183709) | 1.5 | 18WL010 |
| OTU10 | 18WL012 (MT183711) | 0.85 | 18WL012 |
| OTU11 | 18WL013 (MT183712) | 0.55 | 18WL013, 18WL046 |
| OTU12 | 18WL015 (MT183714) | 0.4 | 18WL015 |
| OTU13 | 18WL019 (MT183717) | 4.05 | 18WL019 |
| OTU14 | 18TP376 (MT184043) | 0.9 | 18TP018, 18TP376, 18TP372, 18WL022 |
| OTU15 | 18WL066 (MT183761) | 1.4 | 18WL066, 18WL025 |
| OTU16 | 18WL027 (MT183724) | 0.8 | 18WL027 |
| OTU17 | 18TP140 (MT183983) | 0.7 | 18WL033, 18TP367, 18TP140, 18TP347, 18TP127, 18WL028 |
| OTU18 | 18WL035 (MT183732) | 0.9 | 18WL035 |
| OTU19 | 18WL096 (MT183790) | 1.1 | 18TP395, 18WL096, 18WL036 |
| OTU20 | 18WL038 (MT183734) | 3.2 | 18WL038 |
| OTU21 | 18TP489 (MT184153) | 1.1 | 18TP440, 18TP404, 18TP438, 18TP396, 18TP359, 18TP043, 18TP029, 18TP434, 18TP451, 18TP390, 18TP457, 18TP450, 18TP494, 18TP507, 18TP139, 18TP431, 18TP442, 18TP419, 18TP379, 18WL048, 18TP417, 18TP414, 18TP013, 18WL074, 18WL073, 18TP497, 18TP445, 18TP484, 18TP356, 18TP348, 18TP397, 18TP163, 18TP159, 18TP489, 18TP415, 18TP166, 18TP026, 18TP055, 18TP065, 18WL094, 18TP158, 18TP454, 18TP424, 18TP398, 18TP459, 18WL136, 18TP157, 18TP164, 18WL057, 18TP111, 18TP087, 18WL042 |
| OTU22 | 18WL043 (MT183738) | 2.4 | 18WL111, 18WL062, 18WL128, 18WL129, 18WL067, 18WL065, 18WL134, 18WL080, 18WL063, 18WL125, 18WL106, 18HH015, 18WL043 |
| OTU23 | 18WL097 (MT183791) | 2.4 | 18WL097, 18WL044 |
| OTU24 | 18TP401 (MT184066) | 2.1 | 18TP402, 18TP389, 18TP383, 18TP466, 18TP401, 18WL045 |
| OTU25 | 18WL049 (MT183744) | 3.6 | 18WL049 |
| OTU26 | 18WL054 (MT183749) | 1.3 | 18WL054 |
| OTU27 | 18WL127 (MT183821) | 1.1 | 18WL059, 18WL087, 18WL078, 18WL092, 18WL137, 18WL105, 18WL123, 18WL127, 18WL095, 18WL072, 18WL084, 18WL055 |
| OTU28 | 18WL075 (MT183770) | 3.35 | 18WL075, 18WL069 |
| OTU29 | 18WL071 (MT183766) | 2.05 | 18WL071 |
| OTU30 | 18WL079 (MT183) | 2.55 | 18WL093, 18WL079, 18WL077 |
| OTU31 | 18WL085 (MT183780) | 1.6 | 18WL085 |
| OTU32 | 18WL098 (MT183792) | 4.4 | 18WL098 |
| OTU33 | 18WL099 (MT183793) | 2.9 | 18WL099 |
| OTU34 | 18WL101 (MT183795) | 2.2 | 18WL101 |
| OTU35 | 18WL103 (MT183797) | 4.35 | 18WL103 |
| OTU36 | 18TP437 (MT184101) | 2.3 | 18TP437, 18WL104 |
| OTU37 | 18WL107 (MT183801) | 0.6 | 18WL107 |
| OTU38 | 18TP135 (MT183978) | 1.7 | 18TP135, 18WL109 |
| OTU39 | 18WL112 (MT183806) | 0.4 | 18WL112 |
| OTU40 | 18TP486 (MT184150) | 1.7 | 18WL121, 18TP486, 18WL116 |
| OTU41 | 18WL120 (MT183814) | 1.85 | 18WL120 |
| OTU42 | 18WL122 (MT183816) | 2.25 | 18WL122 |
| OTU43 | 18TP409 (MT184074) | 1.85 | 18TP050, 18TP444, 18TP153, 18TP075, 18TP458, 18TP471, 18TP420, 18TP413, 18TP358, 18TP499, 18TP405, 18TP409, 18TP435, 18TP447, 18TP064, 18WL132, 18WL124 |
| OTU44 | 18WL126 (MT183820) | 1.2 | 18WL126 |
| OTU45 | 18WL130 (MT183824) | 2.4 | 18WL130 |
| OTU46 | 18WL133 (MT183827) | 3.9 | 18WL133 |
| OTU47 | 18WL135 (MT183829) | 2.05 | 18WL135 |
| OTU48 | 18HH002 (MT183832) | 0.8 | 18HH002 |
| OTU49 | 18TP049 (MT183892) | 2.1 | 18TP124, 18TP122, 18TP120, 18TP051, 18TP059, 18TP117, 18TP123, 18TP146, 18TP091, 18TP119, 18TP105, 18TP045, 18TP062, 18TP115, 18TP077, 18TP085, 18TP049, 18TP066, 18TP074, 18TP102, 18TP063, 18TP083, 18TP094, 18TP109, 18TP012, 18TP112, 18TP101, 18TP014, 18TP069, 18TP073, 18TP078, 18TP086, 18TP097, 18TP098, 18TP104, 18TP114, 18TP129, 18TP016, 18TP042, 18TP061, 18TP067, 18TP079, 18TP080, 18TP081, 18TP096, 18TP092, 18TP006, 18TP038, 18TP118, 18TP015, 18TP052, 18TP047, 18TP084, 18TP060, 18TP048, 18TP009, 18TP025, 18HH016, 18TP039, 18TP068, 18TP113, 18TP024, 18TP154, 18HH003, 18TP027, 18TP037, 18TP046, 18TP053, 18TP054, 18TP071, 18TP107 |
| OTU50 | 18HH004 (MT183834) | 0.2 | 18HH004 |
| OTU51 | 18HH012 (MT183842) | 0.45 | 18HH009, 18HH008, 18HH012, 18HH010, 18HH005, 18HH006, 18HH007, 18HH011, 18HH013 |
| OTU52 | 18TP082 (MT183925) | 1.65 | 18TP076, 18TP017, 18TP082, 18TP022, 18TP023, 18TP056, 18TP021, 18TP007, 18TP041, 18TP002 |
| OTU53 | 18TP452 (MT184116) | 1.95 | 18TP452, 18TP010 |
| OTU54 | 18TP439 (MT184103) | 2.8 | 18TP469, 18TP403, 18TP407, 18TP456, 18TP463, 18TP468, 18TP380, 18TP364, 18TP473, 18TP425, 18TP467, 18TP505, 18TP432, 18TP433, 18TP382, 18TP406, 18TP477, 18TP483, 18TP485, 18TP491, 18TP498, 18TP506, 18TP421, 18TP149, 18TP121, 18TP449, 18TP490, 18TP150, 18TP455, 18TP386, 18TP156, 18TP151, 18TP138, 18TP143, 18TP155, 18TP152, 18TP162, 18TP342, 18TP439, 18TP446, 18TP462, 18TP496, 18TP448, 18TP500, 18TP371, 18TP161, 18TP349, 18TP011 |
| OTU55 | 18TP020 (MT183864) | 0.4 | 18TP020 |
| OTU56 | 18TP035 (MT183879) | 0.55 | 18TP036, 18TP035, 18TP031, 18TP030 |
| OTU57 | 18TP033 (MT183877) | 0.55 | 18TP034, 18TP033, 18TP032 |
| OTU58 | 18TP361 (MT184028) | 0.7 | 18TP148, 18TP165, 18TP147, 18TP100, 18TP375, 18TP361, 18TP352, 18TP144, 18TP350, 18TP346, 18TP044 |
| OTU59 | 18TP116 (MT183959) | 0.9 | 18TP160, 18TP090, 18TP093, 18TP116, 18TP099, 18TP089, 18TP088, 18TP103, 18TP057 |
| OTU60 | 18TP134 (MT183977) | 1.5 | 18TP133, 18TP130, 18TP134, 18TP132, 18TP125, 18TP131, 18TP392, 18TP476, 18TP058 |
| OTU61 | 18TP478 (MT184142) | 2.6 | 18TP394, 18TP478, 18TP141, 18TP070 |
| OTU62 | 18TP072 (MT183915) | 2.65 | 18TP072 |
| OTU63 | 18TP095 (MT183938) | 0.45 | 18TP095 |
| OTU64 | 18TP106 (MT183949) | 1.85 | 18TP106 |
| OTU65 | 18TP108 (MT183951) | 1.5 | 18TP108 |
| OTU66 | 18TP110 (MT183953) | 2.45 | 18TP110 |
| OTU67 | 18TP126 (MT183969) | 2.9 | 18TP418, 18TP493, 18TP128, 18TP126 |
| OTU68 | 18TP136 (MT183979) | 0.65 | 18TP136 |
| OTU69 | 18TP137 (MT183980) | 0.9 | 18TP137 |
| OTU70 | 18TP142 (MT183985) | 0.95 | 18TP142 |
| OTU71 | 18TP145 (MT183988) | 2.85 | 18TP145 |
| OTU72 | 18TP345 (MT184013) | 3.75 | 18TP475, 18TP436, 18TP470, 18TP427, 18TP495, 18TP353, 18TP482, 18TP344, 18TP422, 18TP370, 18TP368, 18TP345, 18TP354, 18TP343 |
| OTU73 | 18TP351 (MT184019) | 0.55 | 18TP351 |
| OTU74 | 18TP362 (MT184029) | 2.6 | 18TP362, 18TP365, 18TP357 |
| OTU75 | 18TP363 (MT184030) | 3 | 18TP363, 18TP360 |
| OTU76 | 18TP366 (MT184033) | 2.95 | 18TP366 |
| OTU77 | 18TP369 (MT184036) | 3.1 | 18TP369 |
| OTU78 | 18TP373 (MT184040) | 4.35 | 18TP373 |
| OTU79 | 18TP374 (MT184041) | 0.5 | 18TP374 |
| OTU80 | 18TP377 (MT184044) | 2.65 | 18TP377 |
| OTU81 | 18TP460 (MT184124) | 3.7 | 18TP504, 18TP429, 18TP430, 18TP492, 18TP460, 18TP378 |
| OTU82 | 18TP385 (MT184051) | 2.15 | 18TP385 |
| OTU83 | 18TP503 (MT184167) | 0.6 | 18TP488, 18TP503, 18TP399, 18TP480, 18TP502, 18TP487, 18TP472, 18TP387 |
| OTU84 | 18TP391 (MT184057) | 2.45 | 18TP391 |
| OTU85 | 18TP465 (MT184129) | 4.2 | 18TP465, 18TP400 |
| OTU86 | 18TP408 (MT184073) | 2.4 | 18TP408 |
| OTU87 | 18TP411 (MT184076) | 1.15 | 18TP411 |
| OTU88 | 18TP501 (MT184165) | 4.4 | 18TP501, 18TP412 |
| OTU89 | 18TP416 (MT184081) | 2.05 | 18TP416 |
| OTU90 | 18TP423 (MT184088) | 4.2 | 18TP423 |
| OTU91 | 18TP441 (MT184105) | 0.9 | 18TP441 |
| OTU92 | 18TP443 (MT184107) | 0.3 | 18TP443 |
| OTU93 | 18TP453 (MT184117) | 0.8 | 18TP453 |
| OTU94 | 18TP508 (MT184172) | 4.2 | 18TP508 |
